# Understanding the axonal response to injury by *in vivo* imaging in the mouse spinal cord: A tale of two branches

**DOI:** 10.1016/j.expneurol.2019.04.008

**Published:** 2019-04-12

**Authors:** Binhai Zheng, Ariana O. Lorenzana, Le Ma

**Affiliations:** a Department of Neurosciences, School of Medicine, University of California San Diego, La Jolla, CA, USA; b VA San Diego Healthcare System, San Diego, CA, USA; c Department of Neuroscience, Jefferson Synaptic Biology Center, Vickie and Jack Farber Institute for Neuroscience, Sydney Kimmel Medical College, Thomas Jefferson University, Philadelphia, PA, USA

**Keywords:** In vivo imaging, Spinal cord, 2-photon microscopy, Neuronal responses to axonal injury, Axon regeneration, Axon degeneration, Axonal branches, Bifurcation

## Abstract

Understanding the basic properties of how axons respond to injury in the mammalian central nervous system (CNS) is of fundamental value for developing strategies to promote neural repair. Axons possess complex morphologies with stereotypical branching patterns. However, current knowledge of the axonal response to injury gives little consideration to axonal branches, nor do strategies to promote axon regeneration. This article reviews evidence from *in vivo* spinal cord imaging that axonal branches markedly impact the degenerative and regenerative responses to injury. At a major bifurcation point, depending on whether one or both axonal branches are injured, neurons may choose either a more self-preservative response or a more dynamic response. The stabilizing effect of the spared branch may underlie a well-known divergence in neuronal responses to injury, and illustrates an example where *in vivo* spinal cord imaging reveals insights that are difficult to elucidate with conventional histological methods.

The first *in vivo* optical imaging study in the mouse spinal cord was performed with wild-field fluorescent microscopy, which demonstrated acute axon degeneration and subacute axon regeneration following a small mechanical injury (Kerschensteiner et al., 2005). Since then, a number of studies have been reported using advanced microscopy and especially multi-photon microscopy that either revealed new biological insights and/or propelled technological advances (Bareyre et al., 2011; Davalos et al., 2008; Di Maio et al., 2011; Dray et al., 2009; Erturk et al., 2007; Evans et al., 2014; Farrar et al., 2012; Fenrich et al., 2012; Ran et al., 2016; Sekiguchi et al., 2016; Williams et al., 2014; Ylera et al., 2009). Many of these studies will be covered in this Special Issue. Some of these articles will cover methodological aspects such as surgical preparations, chronic window implants and fluorescent labeling of both neural and non-neural cell types (Cheng et al., this issue; Evans et al., this issue). Here we focus on the biology, and in particular the study that demonstrated the importance of axonal branches in the degenerative and regenerative responses to injury (Lorenzana et al., 2015), which utilized the three primary advantages of *in vivo* imaging we discuss below. We will place this finding in historical context and also discuss potential implications on the basic understanding of the axonal response to injury.

## 1. The advantages of *in vivo* imaging as an experimental paradigm to study the axonal response to injury in the mouse spinal cord

A major incentive to develop *in vivo* optical imaging in the mouse spinal cord as an experimental paradigm was to elucidate the events following injury to the axons (Akassoglou et al., 2017; Laskowski and Bradke, 2013; Misgeld and Kerschensteiner, 2006). Axons in the mammalian central nervous system (CNS) have limited innate ability to regenerate following injury, which to a large extent underlies the permanent paralysis and functional deficits in people with spinal cord injury (He and Jin, 2016; Silver et al., 2015; Sofroniew, 2018). For over a century, generations of neuroscientists have been endeavoring to understand how axons in the CNS respond to injury and how axon regeneration can be enhanced in order to promote functional restoration.

Animal models (especially rodent models) of spinal cord injury have been extensively used to study the axonal response to injury and to investigate the underlying molecular and cellular mechanisms (Lee and Lee, 2013; Zheng et al., 2006). In a typical experiment, at a certain time point after experimental spinal cord injury and other manipulations (e.g. for axon tracing and/or molecular and cellular interventions), mice or rats are killed to collect tissues for histological examination (e.g., to detect axons). While these models are designed to mimic human conditions, there are a few inherent limitations for histological examination of injured spinal cord tissues, referred to here as conventional methods (Table 1).

First, it is difficult, if not impossible, to distinguish regenerating axons from spared axons with conventional methods. Many positive findings in the literature could not be reproduced or substantiated later on (Steward et al., 2012; Steward et al., 2003; Tuszynski and Steward, 2012). While this is partly due to the complexity of such injury models, a major underlying cause is the inability to distinguish regenerating axons from spared ones. Spared axons are often mistakenly construed as regenerating axons, leading to erroneous conclusions. In principle, *in vivo* imaging can bypass this limitation by visually verifying the injury before a regenerative response is assessed.

Second, there is limited temporal resolution with conventional methods. Histological examination of terminal samples only allows for snapshots of a potentially dynamic process. Temporal resolution is lost when only the last time point can be examined. In order to construct a time sequence, a large number of samples need to be collected at many time points, which translate into high cost and low efficiency. A time sequence can only be deduced, but not absolutely established. Individual variations will be overlooked over group behaviors. Even erroneous conclusion can be drawn. For example, an injured axon that is static would be equated to an axon that has undergone both regeneration and retraction so the final outcomes appear similar in both cases. Missed opportunities will arise as a result. The higher temporal resolution of *in vivo* time-lapse imaging allows for a greater ability to detect small changes in axonal morphologies, even though the optical resolution remains the same. For instance, an intervention that elicits small (e.g. ~100–200 μm) but consistent axon regeneration may be masked by the inherently large variability (e.g. ~200–600 μm) in axonal retraction early after injury with conventional methods. By contrast, *in vivo* imaging can resolve this small new axon regeneration by taking into account individual variability in axonal retraction so that a small amount of regeneration may be detected.

Third, it is difficult to isolate experimental variables with conventional methods. Because experimental spinal cord injury typically involves larger complex lesions such as transections, crushes and contusions, a high number of variables may influence the anatomical and functional outcomes. It is often not straightforward to pinpoint which one of the many variables is key to a particular molecular and/or cellular intervention that has impacted the outcome. With *in vivo* imaging, one may introduce a more defined axon injury, e.g. with laser axotomy or a small pinprick, such that certain aspects of traumatic spinal cord injury can be minimized to allow for the investigation of neuron-intrinsic properties in the absence of far-reaching environmental changes. Thus, opportunities exist with *in vivo* imaging to isolate experimental variables and to study them one at a time for their contribution to the final outcome. It should be noted, however, that laser injuries or pin-pricks do not recapitulate clinical conditions of spinal cord injury well, and thus it is important to also study clinically relevant spinal cord injury models (e.g. contusions) with *in vivo* imaging (Williams et al., 2014).

Other differences exist. *In vivo* imaging does not suffer from fixation and sample preparation-induced artifacts, although its implementation in the spinal cord presents its own technical challenges due to heartbeats and breathing movements (Davalos et al., 2008). On the other hand, tried-and-true histological methods are more likely to be robust across different experimental conditions, e.g., in immunohistochemical detections of macromolecules and cellular structures. Furthermore, technological advances in tissue clearing and 3D imaging are giving new life to histological methods (Richardson and Lichtman, 2015; Seo et al., 2016). Thus, histological methods remain the primary approach in characterizing tissue and cellular responses to injury and disease in the CNS, which is complemented by *in vivo* imaging. Readers are referred to excellent reviews by others in this Special Issue for extensive discussions on the challenges of *in vivo* imaging and ways to overcome them.

## 2. Axonal branches are a cardinal feature of neurons

The complexity of the nervous system underlies complex behavior. A cardinal feature of neurons is their complex and often stereotypical axon branching patterns. Axonal branches form an important foundation for information distribution and integration in a functional nervous system. At a minimum, branches allow neurons to connect with multiple synaptic targets at different anatomical locations. Based on their location and morphology, axonal branches can be divided into three major types. The most recognized type is terminal arbors. Located at axonal terminals, these structures have exuberant branches that cover a large area of synaptic targets (Gibson and Ma, 2011). Examples are found in the terminals of retinal ganglion cells, dorsal root ganglion (DRG) sensory neurons, and many motor neurons. More simplified forms of axon branching include collateral branches and bifurcation.

Collaterals are defined as axonal branches emerging from the main axonal shaft. Again, DRG neurons provide a good example, as collateral branches arise from both ascending and descending axons, thereby allowing the sensory neurons from one body location to synapse with motor neurons either directly or indirectly in different spinal cord segments. Another example is the pyramidal neurons in cortical layer II/III, which send their only axons ventrally into the callosal tract to innervate their contralateral targets, and in the meantime also innervate layer II/III and layer V in the ipsilateral side with the formation of collateral branches (Kalil and Dent, 2014).

Axon bifurcation appears as two axons splitting from a common axonal shaft. This is best exemplified by the central projections of the DRG sensory neurons that form the dorsal column in the spinal cord (also see below). There, the axons from the dorsal root split into two, thereby forming ascending branches that synapse with higher order relay neurons in the brainstem and descending branches that often terminate in cauda equina. Although studies in cultured neurons have suggested that axon bifurcation results from growth cone splitting (Acebes and Ferrus, 2000), *in vivo* studies based on mutant phenotypes argue that they may be formed as a special case of collateral branches (Gibson and Ma, 2011).

These different types of branches often have stereotypic patterns, pointing to the critical roles they play in shaping functional neural circuits. Work from multiple laboratories around the world has led to significant advances in understanding the cellular mechanisms of their development. It is now well accepted that developmental axon branching is precisely controlled by both extracellular factors and intrinsic genetic programs, including pathways that are known to regulate axon growth and guidance (Armijo-Weingart and Gallo, 2017; Bilimoria and Bonni, 2013; Gibson and Ma, 2011; Kalil and Dent, 2014; Winkle et al., 2016). It remains to be seen how the knowledge gained on developmental axon branching may be translated to axonal repair after injury in the adult CNS.

## 3. Lack of knowledge on the injury response of axonal branches

**Fig. 1.**
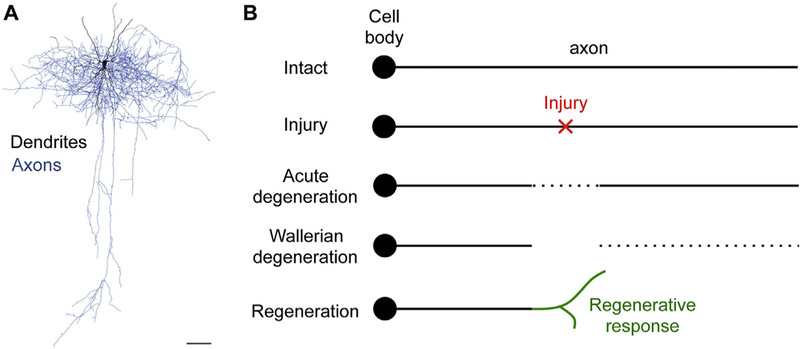
The dichotomy between the complexity of neuronal morphology and the linear model of axon degeneration and regeneration following injury. A) The complexity of neuronal morphology as illustrated by the intricate branching patterns of axons (blue) and dendrites (black) of this layer 2/3 rat barrel cortical interneuron. Modified from (Schmidt and Rathjen, 2010). Scale bar = 100 μm. B) Current textbook illustration of the degenerative and regenerative response of an axon as a linear entity to injury. Myelin and myelinating cells (e.g. oligodendrocytes) are omitted for simplicity. (For interpretation of the references to colour in this figure legend, the reader is referred to the web version of this article.)

While neurons exhibit complex and varied morphologies with intricate and often stereotypical axonal (and dendritic) patterns (Fig. 1A), current neuroscience textbooks typically illustrate an injured axon to begin as a linear entity (Fig. 1B). Following axonal injury, there is an acute phase of bidirectional axon degeneration for a limited distance, as described in the first *in vivo* spinal cord imaging study (Kerschensteiner et al., 2005). This is followed by a later phase of the extensively studied Wallerian degeneration distal to the injury site. The proximal end of the injured axon mounts a regenerative response. In the peripheral nervous system (PNS), this regeneration can often be successful, reaching appropriate synaptic targets and leading to functional recovery. In the CNS, however, this regenerative attempt typically fails, resulting in permanent functional deficits and paralysis, as in spinal cord injury.

The literature has given ample consideration to axon branching as a neural repair mechanism known as sprouting (Geoffroy and Zheng, 2014; Schwab and Strittmatter, 2014; Sofroniew, 2018; Tuszynski and Steward, 2012). Collateral sprouting, or branching from either uninjured axons or injured axons (in the latter case, proximal to the injury site) is a well-known form of axonal repair that could be harnessed to promote functional restoration. However, how the branching patterns of axons prior to injury impact their injury response is usually not considered. Much of the textbook knowledge on Wallerian degeneration and axon regeneration originated from studies of peripheral nerve injury, where there is minimal branching along the uninjured nerve. What happens when branched axons are injured, which likely constitute the majority of cases when CNS axons are injured? This complexity also applies to dendrites but we focus on axons here.

## 4. Dorsal column sensory axons as a model to examine branch structure in the axonal response to injury

**Fig. 2.**
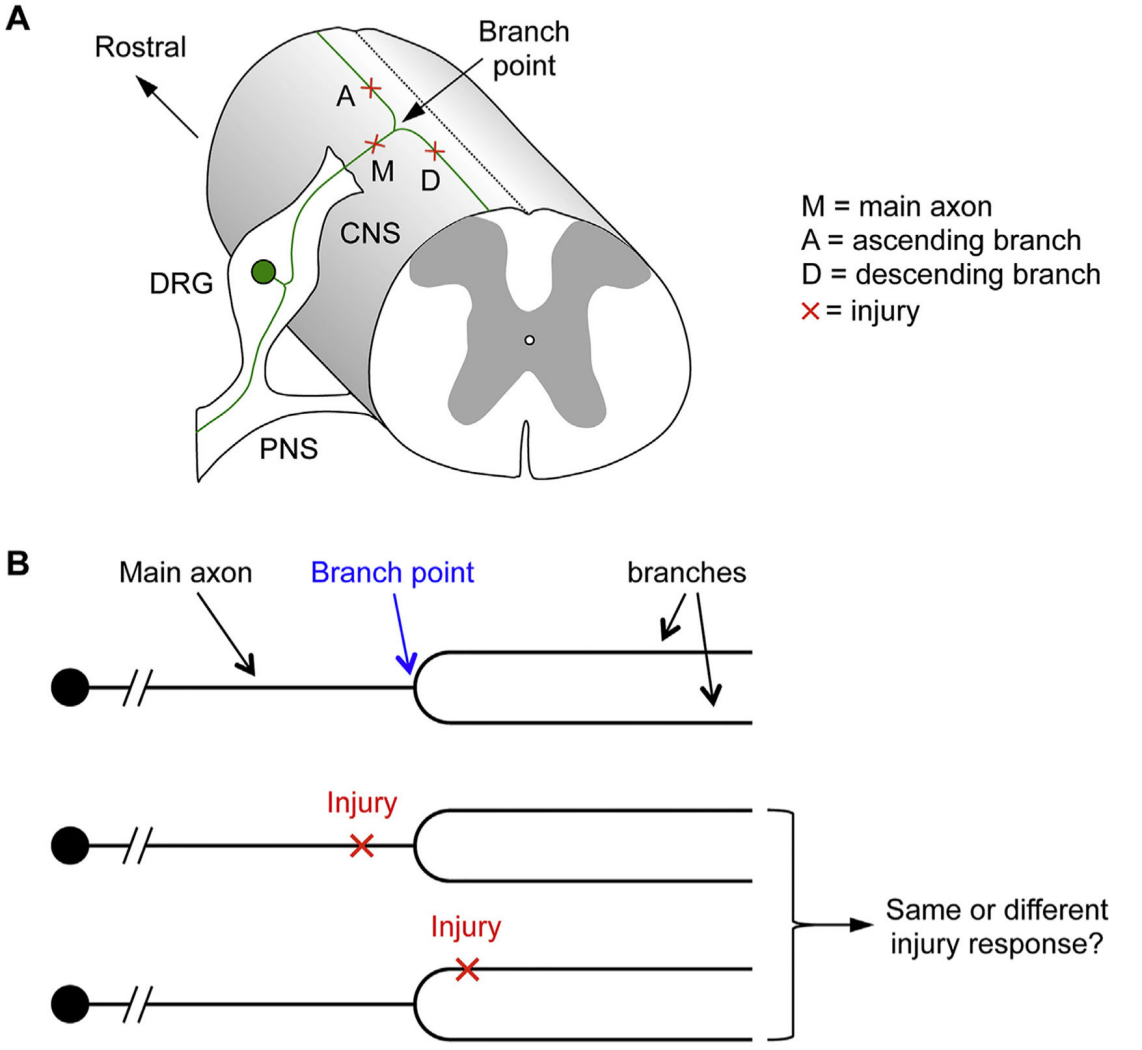
Using dorsal column sensory axons as a model to study the effect of branch structure on the axonal response to injury. A) The secondary branch or bifurcation point (marked as “Branch Point) of dorsal column sensory axons gives rise to an ascending branch and a descending branch in the CNS. DRG, dorsal root ganglion. Red cross marks the location of a possible laser injury. B) A laser injury can be directed just before or after the branch point, with axonal responses assessed with *in vivo* imaging to determine if the injury location relative to the branch point impacts the outcome, and if so, how. (For interpretation of the references to colour in this figure legend, the reader is referred to the web version of this article.)

Dorsal column sensory axons serve as an excellent model to investigate branch structure in the axonal response to injury (Fig. 2A) (Lorenzana et al., 2015). The cell bodies of these axons reside in the dorsal root ganglia (DRG) outside of the spinal cord. A primary axon bifurcates into a peripheral branch that innervates the peripheral organs (skin, muscle etc.), while the central branch enters the spinal cord via the dorsal root entry zone (DREZ). Once inside the spinal cord, the central branch bifurcates again, with an ascending branch extending up the spinal cord towards the brain and a descending branch that synapses in the spinal cord. It is at this secondary bifurcation point that a highly localized laser injury can be precisely directed at the axon to assess the effect of the injury location on the outcome (Fig. 2A). Specifically, an injury can be applied either just before or just after the branch point to assess the effect of the injury location relative to the branch point with *in vivo* imaging (Fig. 2A, B). These injury locations can be kept well within the CNS so that the primary differences among them are their relative positions in reference to the branch point. It is conceivable that when the main axon is injured, both the ascending and descending branches will degenerate sooner or later due to Wallerian degeneration. When the ascending or descending branch is injured close to the branch point, it is less predictable what will happen to the injured branch, the branch point and the remaining uninjured branch (Fig. 2B). This question would be difficult to assess with conventional experimental spinal cord injury and histological methods because it is difficult to precisely pinpoint (or pre-determine) the injury location along a particular axon. An *in vivo* imaging paradigm offers the opportunity to guide a highly localized injury to a precise location and to visualize the axon over time before and after injury.

## 5. Branch structure impacts the degenerative and regenerative response of injured axons

Equipped with a femtosecond two-photon laser, one can localize an injury to sever one fluorescently labeled axon at a time with *in vivo* imaging. Such highly localized injuries allow for the effect of the injury location along the axon to be assessed with a high degree of accuracy and precision. Specific axonal injuries can be verified visually before the axonal responses are followed at acute (minutes to hours), subacute (days) and more chronic time points (weeks to months) (Lorenzana et al., 2015). Observations at the subacute or later time points require the re-opening of the imaging site, unless a chronic window is implanted for repetitive long term follow-up (Farrar et al., 2012; Fenrich et al., 2012) (see article by Schaffer and colleague in this issue).

The initial incentive to localize the laser injury near a branch point was simply to use the branch point as a convenient fiducial marker in order to facilitate the identification of the injury location at later time points. Branch patterns and morphologies remain relatively stable over an extended period of time even considering the immune response that is unavoidable with invasive optical imaging. However, it quickly became clear that the injury location relative to the bifurcation point would be an important scientific variable to investigate (Lorenzana et al., 2015). At the time, Kerschensteiner et al. had reported the bidirectional acute axon degeneration minutes and hours after a pinprick lesion (Kerschensteiner et al., 2005). The same bidirectional axon degeneration was seen with laser injury (Lorenzana et al., 2015). Axon degeneration, in the form of fragmentation and to a lesser degree retraction, propagated from the laser injury site bidirectionally for hours after injury. This degeneration subsided a few hours after laser injury, but often continued for a limited extent days after injury. Over time, anterograde Wallerian degeneration expectedly took over and eliminated the distal axonal segment. Temporally, the distinction between the bidirectional acute degeneration and the unidirectional Wallerian degeneration was not clear cut (Lorenzana et al., 2015). Regardless, within days after injury, axon regeneration could be observed by *in vivo* imaging that consists of both branching and elongation.

**Fig. 3.**
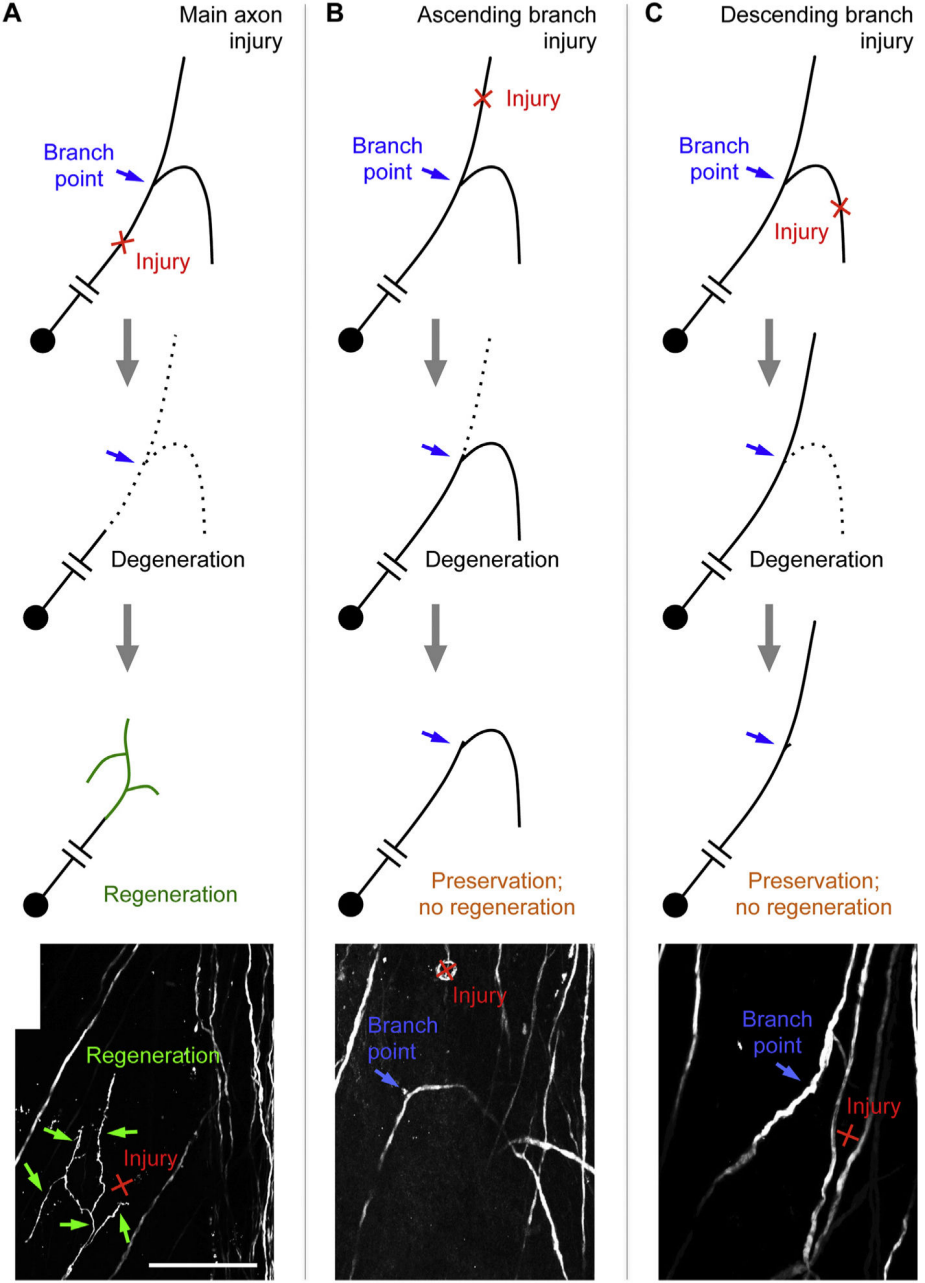
Injury location relative to a bifurcation point impacts the degenerative and regenerative responses of axons. A) Main axon injury leads to the elimination of both branches followed by a regenerative response as detected by *in vivo* imaging. B, C) Ascending (B) or descending (C) axon injury close to the branch site leads to near complete elimination of one branch by both retrograde and anterograde degeneration, leading to the preservation of the spared branch but no detectable regenerative response from the injured branch as assessed by *in vivo* imaging. In (A–C), the bottom panel, adapted from (Lorenzana et al., 2015), shows an image acquired by *in vivo* imaging that illustrates the typical outcome for each injury location five days after injury. Scale bar = 100 μm.

Strikingly, the injury location relative to the (secondary) bifurcation point had a dramatic effect on the ensuing degenerative and regenerative responses following a laser-induced axonal injury (Lorenzana et al., 2015). At both the acute (hours after injury) and subacute (5 days after injury) time points, retrograde degeneration after an ascending or descending branch injury close to the branch point was typically blocked from further progression when encountering the bifurcation point, often leaving a short stub (Fig. 3B, C). Retrograde degeneration rarely breached the branch point. When it did, the other branch also degenerated as expected. This was in direct contrast to the anterograde degeneration, as in the case of main axon injury, which was not influenced at all by the presence of a branch point even at acute time points (Fig. 3A). Thus, the branch point appeared to serve as a barrier for retrograde but not anterograde degeneration (Lorenzana et al., 2015).

The next obvious question was how axon regeneration is impacted by the branch structure? After main axon injury, most axons (~90%) exhibited a regenerative response (a combination of branching and elongation) as detected by *in vivo* imaging 5 days after injury (Lorenzana et al., 2015). This regenerative response dramatically declined to ~10% when the ascending or descending branch was injured close to the branch point. In most cases, retrograde degeneration proceeded to the branch point without breaching it, followed by no detectable regeneration. In each of the rare cases where retrograde degeneration breached the branch point, regeneration ensued. Double branch injury also led to a high rate of regeneration (~70%), indicating that it is not the branch point itself, and rather the presence of a spared branch that suppresses regeneration. Taken together, spinal cord *in vivo* imaging enabled the discovery that the presence of a spared axonal branch suppresses the spread of retrograde degeneration from the injured branch as well as the latter’s regeneration, thus stabilizing the remaining axon structure (Lorenzana et al., 2015).

## 6. Precedents in the literature

It turned out that there have been precedents for an axonal branch suppressing the regeneration of an injured branch. In C. elegans, the effect of the injury location relative to a branch point on axon regeneration has been reported for multiple neuronal subtypes also using laser injury and *in vivo* imaging, providing evidence that a spared synaptic branch actively suppresses the regeneration of the injured branch (Wu et al., 2007). Thus, the suppressive effect of a spared axonal branch on the regeneration of an injured branch appears to be phylogenetically conserved.

Another homologous phenomenon may be the process of synaptic branch elimination at the developing neuromuscular junctions. During development, motor axons initially innervate many more muscle fibres than they do in the adult. The excessive connections are permanently removed by extensive pruning of terminal branches after birth so that in the end each muscle fibre is innerved by only one motor neuron (Gan and Lichtman, 1998; Tapia et al., 2012). Terminal rather than proximal branches selectively degenerate, are absorbed into the main axon, and do not regenerate. The total amount of axoplasm does not decrease, reflecting a redistribution of axonal resources (Tapia et al., 2012).

Within the mammalian CNS, it was Cajal who made the first reference to terminal branch elimination following injury, along with other, more well known phenomena (Ramón y Cajal, 1928). By examining histological samples following experimental spinal cord injury, Cajal first described the retraction bulbs, widely considered a hallmark of CNS regeneration failure. However, he also described other, lesser-known axonal responses to injury. Using histological samples 1–1.5 h after experimental spinal cord injury in cats, Cajal described what we know today as acute axon degeneration, which was conclusively demonstrated with *in vivo* imaging (Kerschensteiner et al., 2005). Cajal clearly distinguished this bidirectional acute phase degeneration from Wallerian degeneration (Ramón y Cajal, 1928): “One must distinguish here two kinds of degeneration, Wallerian or secondary, which occurs relatively late and is produced in all the fibres that are separated from their trophic centre; and traumatic degeneration, which is extremely rapid, and was first described by Schiefferdecker; this extends to a variable, but always small distance, from the lips of the wound, in the distal as well as in the proximal stumps.”

**Fig. 4.**
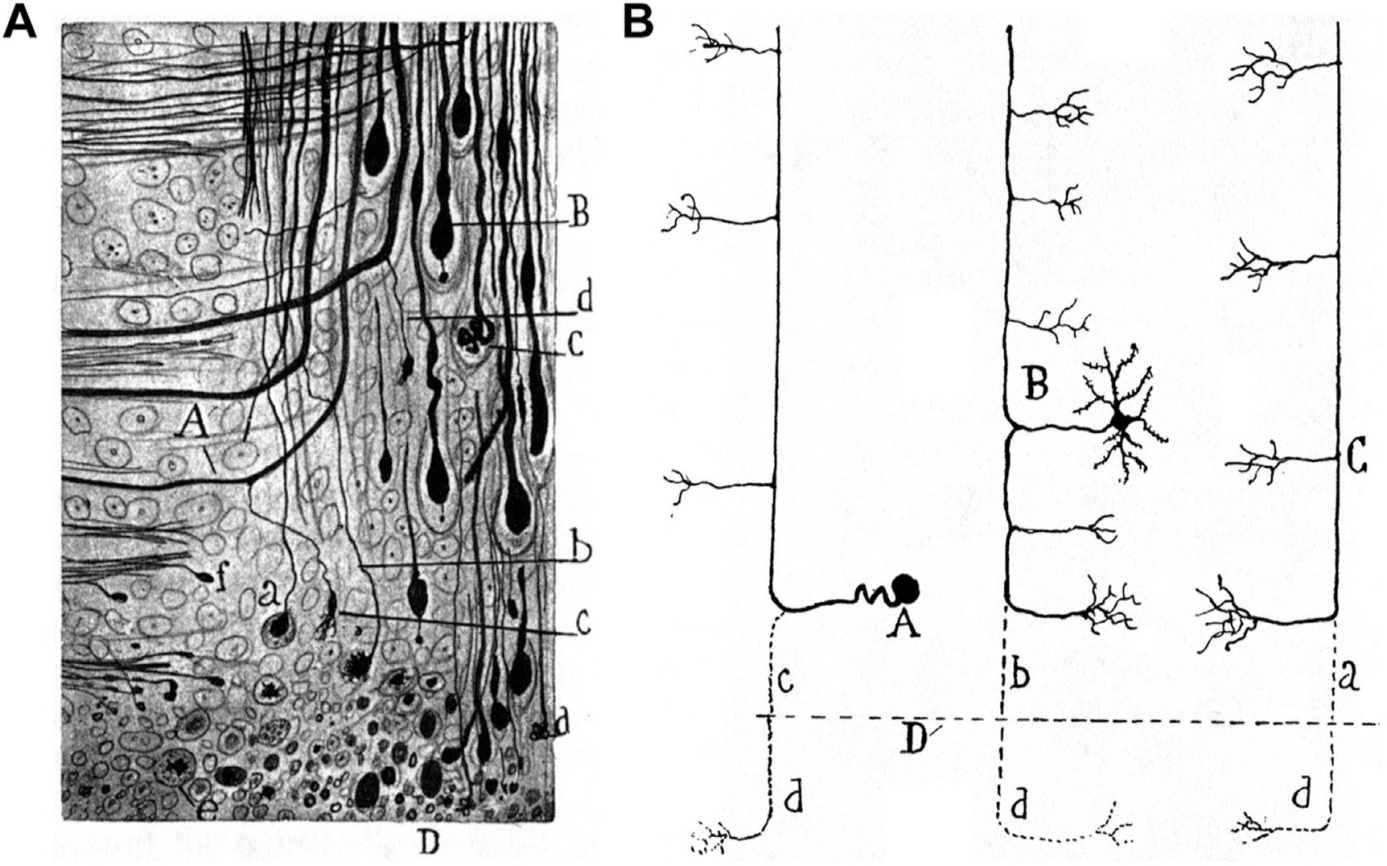
Cajal’s description of terminal branch elimination following CNS injury using classical histological methods. A) Fig. 196 from Cajal’s book. “Piece of the central stump of the spinal wound of a young cat, three days after the operation. A, thickened collaterals which will be transformed into terminal fibres; a, b, c, longitudinal portion of axons destined to disappear; B, club with an appendix; C, final glomerulus; D, edges of the wound with axonic and lipoid detritus; e, free balls which are becoming hya-line.” B) Fig. 195 from Cajal’s book. “Schematic drawing designed to show the resorbed portion of the mutilated conductors of the white matter. A, Fibre of the posterior or sensory fasciculus; B, fibre in continuity with the axon of a funicular neurone; C, fibre in continuity with the axon of a neurone situated in superior centres (pyramidal tract of the cerebrum, etc.); D, plane of the wound; a, b, and c, segments which have disappeared.” Adapted from (Ramón y Cajal, 1928).

Likewise, based on case studies of terminal samples after experimental spinal cord injury (3 days after injury in a cat, see Fig. 4A; also 6 days after injury in a dog and 1.5 months after injury in a rabbit), Cajal made the observation on the complete elimination of an injured branch (Ramón y Cajal, 1928): “But the *most important* change, to which we have already alluded, is the total transformation near the wound, of the axons into arciform fibres which penetrate into the grey matter. It is impossible to see in these regions, in the course of axons coming from the spinal horns or the posterior root, the well-known bifurcation into an ascending and a descending branch. All these conductors, as they encounter the fasciculi, are simply deflected so as to become longitudinal and ascendant if one is dealing with the proximal spinal segment, descendent if one is dealing with the distal segment”. He even extended this observation to multiple neuronal types (Fig. 4B).

The fact that Cajal considered the complete elimination of the terminal branch “the most important change” was likely due to his thinking on the utilitarian nature of this phenomenon (Ramón y Cajal, 1928): “This interesting process of simplification, followed by a compensatory hypertrophy, shows us that traumatic degeneration represents a curious mechanism of reaction of an exquisitely economical and utilitarian character. Thanks to it, nature gets rid, so to speak, of useless mouths of protoplasmic segments that serve no useful purpose.” Compared with Cajal’s case studies on terminal histological samples, *in vivo* imaging allowed for a definitive demonstration of the degenerative process and, more importantly, illuminated the ensuing regenerative response (Lorenzana et al., 2015).

There is one notable difference between the study by Cajal and the in vivo imaging study. While Cajal observed complete elimination of the injured branch even when the injury was localized at some distance away from the branch point, in the *in vivo* imaging study this was observed only when the laser injury was relatively close to the branch point. When the laser injury was slightly moved away from the branch point, retrograde degeneration did not extend close to the branch point, followed by an intermediate regenerative response (Lorenzana et al., 2015). Whether this difference reflected the different injury severities between the two studies (a more traumatic injury in the Cajal study and a very limited laser injury in the *in vivo* imaging study) remains to be tested. It is conceivable that with a more traumatic injury, the initial intrinsic axon degenerative response would be followed by an environment-mediated secondary degenerative response (Evans et al., 2014), leading to the complete branch elimination even when the injury is located at a distance from the branch point. Nevertheless, the data from the *in vivo* imaging study suggest a tug-of-war between the stabilizing, anti-regenerative effect of the spared branch and a pro-regenerative effect of the remaining segment of the injured branch (Lorenzana et al., 2015). Future studies are required to systematically examine the effect of the distance between the injury location and the branch point (especially at greater distances) on the degenerative and regenerative outcomes.

## 7. The synaptic suppression hypothesis and a graded response to axonal injury

**Fig. 5.**
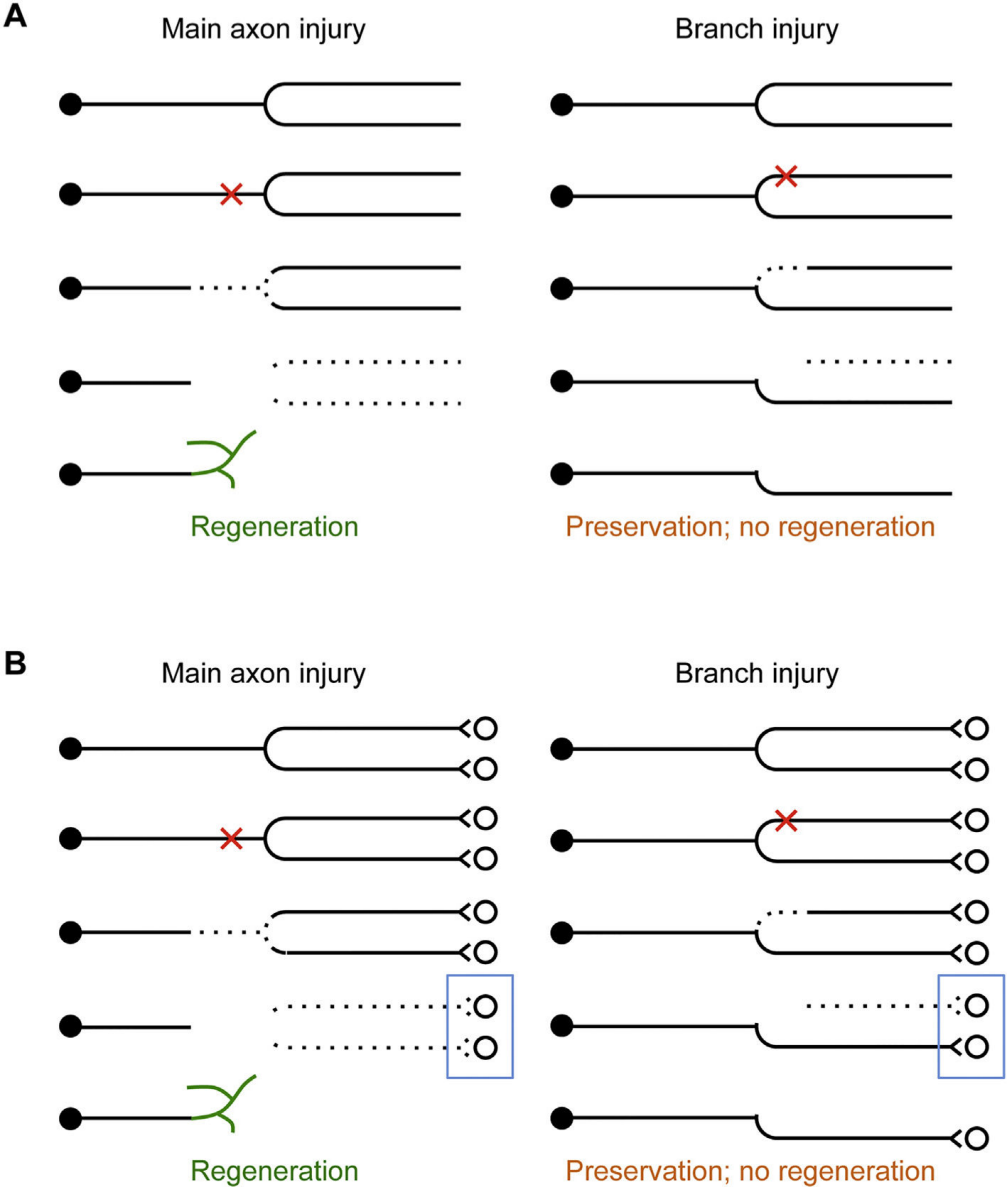
The stabilizing effect of the spared axonal branch and the synaptic suppression hypothesis of axon regeneration. A) Diagram of key observations on axonal responses to branch injury from (Lorenzana et al., 2015). When an axon is injured just before a major bifurcation point, both branches will degenerate sooner or later, and the proximal end mounts a regenerative response that is detectable by *in vivo* imaging. When an axon is injured right after a major bifurcation point so that only one branch is injured, the injured branch will be eliminated for the most part by retrograde (acute and subacute) degeneration and anterograde (Wallerian) degeneration. In most cases, retrograde degeneration does not breach the bifurcation point so the other branch and the main axon are preserved, and no regenerative response is detected by *in vivo* imaging. B) Same as(A) but with synaptic partners drawn to illustrate the synaptic suppression hypothesis of axon regeneration. Maintaining synaptic output may help preserve the remaining axon structure by suppressing both the expansion of degeneration into the spared branch and the regeneration of the injured branch. Blue boxes highlight the fact that no synaptic output remains following main axon injury while some synaptic output remains following branch injury. Note that this is a simplified working model when the injury location is close to the branch point. When the injury location is moved further away from the branch point, the remaining segment of the injured branch may exert a destabilizing effect, counteracting the stabilizing effect of the spared branch. See text for details. (For interpretation of the references to colour in this figure legend, the reader is referred to the web version of this article.)

As discussed above, current textbooks typically illustrate an injured axon as a linear entity even though axons are never purely linear (Fig. 1). With *in vivo* imaging data using dorsal column sensory axons as a model, the considerations presented in this review strongly suggest that any future textbook illustration of axon injury response would benefit from the consideration of axon branching patterns (Fig. 5A). When the main axon is injured (i.e. injury occurs just before a major bifurcation point), both branches will undergo degeneration, which is followed by a regenerative response that is detectable with *in vivo* imaging. A similar response occurs when both branches are injured separately. When only one of the two branches is injured close to the bifurcation point, retrograde degeneration is often limited to the injured branch and does not propagate to the spared branch. This is followed by a lack of a detectable regenerative response, resulting in the stabilization of the remaining axon structure (Lorenzana et al., 2015). Thus, the spared branch appears to stabilize what is left after injury to the other branch.

This stabilizing effect of a spared branch after injury can be best rationalized when synaptic partners are considered (Fig. 5B). When the main axon is injured (or both branches are injured simultaneously), both branches will be eliminated sooner or later. The loss of major synaptic contacts may prompt the neuron to mount a relatively strong regenerative response. When only one of the two branches is injured, the retention of significant synaptic contacts may prompt the neuron to allocate resources to preserve the remaining branch instead of mounting a detectable but futile regenerative response so that some function is maintained.

There are several possible cellular mechanisms by which retrograde degeneration is blocked at the branch point. Cytoskeleton organization at the branch point differs from that in the axonal shaft, which may provide the primary stabilizing effect (Armijo-Weingart and Gallo, 2017; Gallo, 2011). For example, microtubules are usually bundled in axons but splayed at branch points (Ketschek et al., 2015); and some microtubule-associated proteins (e.g. MAP7) are enriched at branch junctions (Tymanskyj et al., 2018). Also, mitochondria are often anchored at the branch sites, providing additional support for branch stabilization (Kiryu-Seo and Kiyama, 2019; Smith and Gallo, 2018; Spillane et al., 2013). Although less evident, other intracellular organelles (e.g. endoplasmic reticulum) and membrane trafficking may also be at play (Winkle et al., 2016).

The suppressive effect of a spared branch on the regeneration of the injured branch (Lorenzana et al., 2015) is consistent with reports in the literature where synaptic or synaptic-like contacts entrap or immobilize regenerating axons, thus inhibiting further regeneration (Di Maio et al., 2011; Filous et al., 2014). Synaptic contacts of the remaining branch may feed back to the cell body to suppress a regenerative response. In the most simplistic scenario, the process of synaptic transmission alone may suppress regeneration. This can be tested by observing the effect of inhibiting synaptic transmission on the regeneration of the injured branch following branch axotomy. Indeed, there is molecular evidence in the literature linking synaptic transmission and the suppression of axon regeneration. Overexpressing the Alpha2delta2 subunit of the voltage-gated calcium channels, a modulator of synaptic transmission (Hoppa et al., 2012), suppresses peripheral axon regeneration *in vivo* (Tedeschi et al., 2016). Conversely, pharmacological inhibition of the Alpha2delta2 subunit weakens synaptic transmission and promotes dorsal column sensory axon regeneration after spinal cord injury (Tedeschi et al., 2016). These data support the synaptic suppression hypothesis of axon regeneration (Meves and Zheng, 2016). It is conceivable that synaptic transmission may also suppress the degeneration of the spared branch, preventing the invasion of the degenerative process initiated from the injured branch. Regardless of whether synaptic contact is sufficient or active synaptic transmission is required to suppress regeneration, retrograde molecular signaling may be an important element. However, rather than a typical retrograde signal from an injured branch that promotes regeneration (Rishal and Fainzilber, 2014), this would involve a growth inhibitory signal originating from the spared branch to suppress regeneration.

**Fig. 6.**
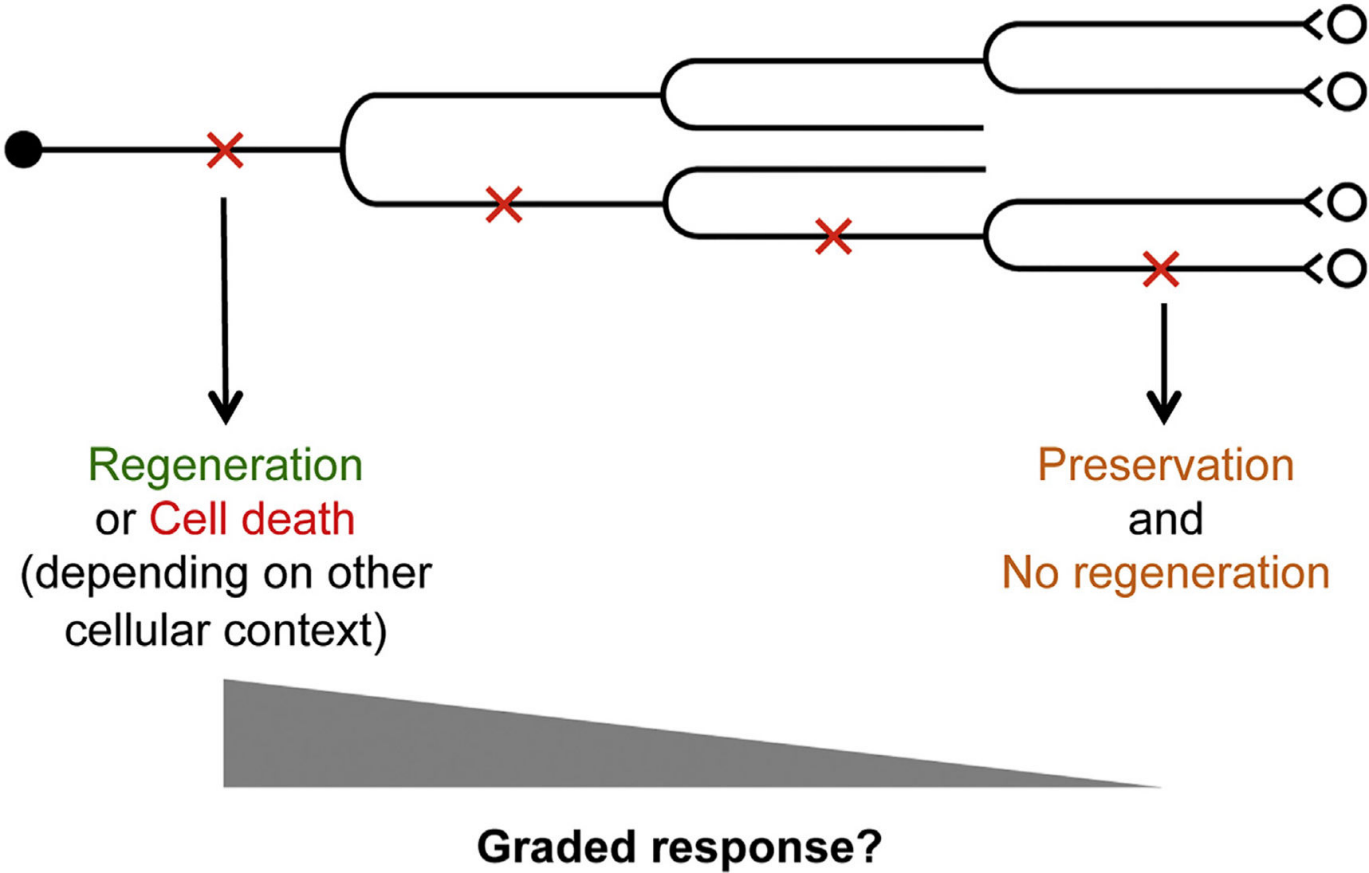
A working model on the graded response to axonal injury. For axons that have multiple orders of branches, an injury to the primary, secondary, tertiary etc. branch would elicit a response of different strengths. Injury to a lower order branch would generally elicit a stronger response as compared with a higher order branch. A strong response could be a strong regenerative response or cell death, depending on other cellular context. A weak response would be self-preservation without a detectable regenerative response. A spectrum of intermediate responses are possible.

On a broader perspective, the stabilizing effect of a spared branch on the axonal response to injury may help explain some well-known phenomena in the field of CNS injury and repair. Given the ordered structure of axon branching, it is conceivable that neurons exhibit a graded response to axonal injury depending on the order of the branch that is injured (Fig. 6). At one end of the spectrum, optic nerve injury, often applied close to the cell bodies, is known to elicit a high level of cell death in retinal ganglion cells. This may represent a drastic response where no or few axonal branches have been spared. Indeed, both axon regeneration and cell death may exemplify a strong injury response. The choice between these two apparently divergent responses may depend on certain intrinsic state of the neurons. Molecular evidence for this context-dependent response came from studies showing that Dual Leucine zipper-bearing Kinase (DLK, or MAP3K12), an important regulator of neuronal responses to injury, promotes both axon regeneration and cell death in retinal ganglion cells (Watkins et al., 2013).

At the other end of the spectrum, a very limited, self-preservative response occurs when the very terminal branch of a complex neuron is injured (Fig. 6). Many intermediate responses are possible depending on the branch structure and, directly or indirectly, the distance to the cell body (Fig. 6). The observation that following spinal cord injury, corticospinal neurons for the most part do not exhibit significant cell death (Nielson et al., 2010; Nielson et al., 2011) is likely due to the fact that a typical spinal cord injury would spare other axonal branches proximal to the injury site, especially those in the brain. Indeed, when the injury is placed much closer to the cell bodies in the brain, significant corticospinal neuron death occurs (Giehl and Tetzlaff, 1996; Hollis II et al., 2009). Furthermore, this proximal axotomy-induced cell death can be rescued by the delivery of neurotrophic factors such as BDNF and IGF-1, indicating a role for trophic factors in cell survival (Giehl and Tetzlaff, 1996; Hollis II et al., 2009). Taken together, the branch structure prior to axonal injury likely has widespread and profound impact on the neuronal response to injury, much of which remains to be discovered.

## 8. Concluding remarks

Data from *in vivo* spinal cord imaging have demonstrated that axonal injury relative to a branch point significantly impacts the degenerative and regenerative response such that a spared branch stabilizes the remaining axon structure. The exact mechanisms underlying the stabilizing effect of a synaptic branch await future investigation. It is conceivable that both synaptic activities and retrograde signaling may be at play. Further studies are required to understand the commonalities and differences among different types of axonal branches (bifurcation, collaterals and terminal arbors) on how they impact injury responses. Regardless, *in vivo* imaging, as has been illustrated in other contributions of this special issue, will continue to provide new biological insights on the neuronal response to axonal injury.

## Figures and Tables

**Table 1:** The three major advantages of *in vivo* imaging as an experimental paradigm to study the axonal response to injury in the mouse spinal cord as compared with conventional histological methods. See text for details.

	Methods	
Features to compare	Conventional histology	In vivo optical imaging
Distinguishing regenerating axons from spared axons	−	+
High temporal resolution; resolving small changes	−	+
Isolating experimental variables	−	+
